# Expanded syringe exchange programs and reduced HIV infection among new injection drug users in Tallinn, Estonia

**DOI:** 10.1186/1471-2458-11-517

**Published:** 2011-06-30

**Authors:** Anneli Uusküla, Don C Des Jarlais, Mart Kals, Kristi Rüütel, Katri Abel-Ollo, Ave Talu, Igor Sobolev

**Affiliations:** 1 Department of Public health, University of Tartu, Ravila street, 50411 Tartu, Estonia; 2 Edmond de Rothschild Chemical Dependency Institute, Beth Israel Medical Center, West 57th Street, New York, NY 10019, USA; 3 Department of Infectious Diseases and Drug Prevention, National Institute for Health Development, Hiiu street, 11619 Tallinn, Estonia; 4 Estonian Drug Monitoring Centre, National Institute for Health Development, Hiiu street, 11619 Tallinn, Estonia; 5 NGO Convictus, Syringe exchange project, Mardi. 10145 Tallinn, Estonia

## Abstract

**Background:**

Estonia has experienced an HIV epidemic among intravenous drug users (IDUs) with the highest per capita HIV prevalence in Eastern Europe. We assessed the effects of expanded syringe exchange programs (SEP) in the capital city, Tallinn, which has an estimated 10,000 IDUs.

**Methods:**

SEP implementation was monitored with data from the Estonian National Institute for Health Development. Respondent driven sampling (RDS) interview surveys with HIV testing were conducted in Tallinn in 2005, 2007 and 2009 (involving 350, 350 and 327 IDUs respectively). HIV incidence among new injectors (those injecting for < = 3 years) was estimated by assuming (1) new injectors were HIV seronegative when they began injecting, and (2) HIV infection occurred at the midpoint between first injection and time of interview.

**Results:**

SEP increased from 230,000 syringes exchanged in 2005 to 440,000 in 2007 and 770,000 in 2009. In all three surveys, IDUs were predominantly male (80%), ethnic Russians (>80%), and young adults (mean ages 24 to 27 years). The proportion of new injectors decreased significantly over the years (from 21% in 2005 to 12% in 2009, p = 0.005). HIV prevalence among all respondents stabilized at slightly over 50% (54% in 2005, 55% in 2007, 51% in 2009), and decreased among new injectors (34% in 2005, 16% in 2009, p = 0.046). Estimated HIV incidence among new injectors decreased significantly from 18/100 person-years in 2005 and 21/100 person-years in 2007 to 9/100 person-years in 2009 (p = 0.026).

**Conclusions:**

In Estonia, a transitional country, a decrease in the HIV prevalence among new injectors and in the numbers of people initiating injection drug use coincided with implementation of large-scale SEPs. Further reductions in HIV transmission among IDUs are still required. Provision of 70 or more syringes per IDU per year may be needed before significant reductions in HIV incidence occur.

## Background

The estimated number of adults and children living with HIV in Eastern Europe and Central Asia rose to 1.5 million in 2008, a 66% increase from 0.9 million in 2001. Three countries in the region (Estonia, Russian Federation and Ukraine) have adult (15-49) HIV prevalence that exceeds 1% [1]. Injecting drug use remains the primary mode of HIV transmission in the region [1,2]. Although coverage with HIV prevention, treatment and care services (syringe exchange programs (SEPs), opioid substitution therapy (OST) and antiretroviral therapy (ART)) for injecting drug users (IDUs) remains low in the region, scattered progress has been reported in expanding SEP and OST programs [3]. Availability of SEP, OST programs and ART and government policies supporting these programs varies widely across the regions. It is estimated that in Eastern Europe an average of 9 needles/syringes are distributed by injection drug user (IDU) per year (ranging from 4 in Russia to 151 in Czech Republic), 1% of IDUs receive OST (ranging from 1% in Georgia and Belarus to 20% in Hungary), and 1% of HIV infected IDUs receive ART (ranging from <1% in Russia to 81% in Czech Republic) [3].

There is considerable evidence that HIV-prevention programs for IDUs, particularly combined programming, in which multiple programs are provided, can be effective in reducing injection-related HIV transmission [4,5]. In many areas in industrialized countries, it has been possible to keep prevalence low indefinitely, literally preventing HIV epidemics among IDUs [6-9]. It is also possible to "reverse" large-scale HIV epidemics among IDUs (i.e. to greatly reduce both HIV incidence and prevalence) with large scale prevention efforts applied over long periods [10-13]. However, there has been relatively little research, other than pilot studies, on the effects of syringe exchange in developing/transitional countries. It is therefore uncertain whether the interventions used in developed countries will have the same degree of effectiveness in transitional countries where these interventions might operate in a hostile policy environment.

Data on new injectors (i.e. people who have started to inject drugs recently) can provide critical insights into the dynamics of an ongoing HIV epidemic among IDUs. First, the proportion of new injectors provides information about the growth of the population at risk of HIV. Second, in epidemics that are driven by injecting drug use with little sexual transmission, all or almost all IDUs will be HIV seronegative when they begin injecting [14,15]. In these situations HIV prevalence among new injectors can be used to estimate HIV incidence. Third, new injectors are often the most difficult subgroup to reach with HIV prevention services -- possibly because they do not identify themselves as drug injectors [16] so that information on whether new injectors are using HIV prevention services can be a good measure of the reach of prevention services.

In this report, we examine trends in new injectors in the IDU population in Tallinn, Estonia from 2005 to 2009 in relation to the expansion of harm reduction services during the same time period.

Estonia has experienced a concentrated HIV epidemic among IDUs, with the highest per capita HIV prevalence in Eastern Europe. There are an estimated 10,000 IDUs (approximately 70% of IDUs in the country) in Tallinn, the capital and largest city [17]. Studies in Estonia have shown a high prevalence of HIV (40-90%) [18,19], and estimated the incidence of HIV in the IDU population >20/100 person years at risk [20]. Based on data from surveys conducted among IDUs in Tallinn [18,19,21], a shift in use from heroin and home-made opiates to fentanyl analogues and amphetamine has occurred during the current decade.

Estonia's capacity to manage its response to HIV and AIDS has increased greatly over the past five years, particularly through funding from the Global Fund to fight HIV/AIDS, tuberculosis, and malaria [22]. Global Fund support was also instrumental in building capacity for the governmental and nongovernmental sectors to interact constructively with each other. This included establishing systems for channeling funds through government to NGOs and mechanisms for dialogue [23]. Amongst other activities, reducing the risk of harm faced by IDUs by scaling-up syringe exchange programs and drug treatment were implemented. The National Institute for Health Development (NIHD) is an agency of the Ministry of Social Affairs and is responsible for implementing and monitoring the majority of the state prevention programs (including those targeting HIV/AIDS and drug use). Detailed descriptions of the HIV epidemic in Estonia and response to the epidemic can be found elsewhere [24]. SEPs were initiated in Estonia in 1997, in Tallinn. By 2009 in Tallinn, 3 organizations were providing SEP through 3 stationary centers (and 7 outreach locations) with 63,333 registered visits (1 center, 2341 visits in 2003; 2 centers, 30,863 visits in 2005) (Table 1) [25]. Methadone detoxification has been available in Estonia since 1998, but opioid substitution maintenance treatment with methadone was officially introduced in 2001 [24]. By the end of 2009 in Tallinn 3 institutions (3 centers) were providing opioid substitution treatment (Table 1) [25].

**Table 1:** Volume of harm reduction services provided in 2003 to 2009 in Tallinn, Estonia

**Year**	**2003**	**2004**	**2005**	**2006**	**2007**	**2008**	**2009**
							
No of syringes distributed	18,010	129,093	230,409	443,961	600,021	734,954	774,782
							
No of condoms distributed throughout SEPs	16,427	76,004	83,975	134,837	158,164	156,735	131,162
							
No of positions for methadone treatment		46	103	183	200	191	209

In Estonia, during the past 10 years the number of patients on ART has increased from 27 in 2000 to 1751 at the end of 2010 [personal communication, Mr. Mihkel Rääk, Ministry of Social Affairs, Estonia, 24.01.2011]. However, only 5-12% of HIV-infected IDUs have reported currently receiving ART [26].

## Methods

Cross sectional studies in 2005, 2007 and 2009 were conducted to assess the prevalence of HIV and risk behavior among IDUs in Tallinn, Estonia. In each study, current IDUs were recruited for an interviewer-administered risk behavior survey covering demographics, drug use history, and HIV risk behavior. HIV counseling was provided and a blood sample was collected for HIV testing. Participants had to: be 18 years or older, be Russian or Estonian speakers, have injected drugs in the previous two months (one month in 2005), and be able to provide informed consent.

Respondent driven sampling (RDS) [18,19] was used in all three surveys. Recruitment began with the non-random selection of 5 - 6 'seeds' representing diverse IDU types (by gender, ethnicity, main type of drug used, and HIV status). Eligible participants received coupons for recruiting up to three of their peers. Coupons were uniquely coded to link participants to their survey responses and biological specimens and for monitoring who recruited whom. Participants who completed the study received a primary incentive (a food coupon worth $ 10) for participation in the study and a secondary incentive (a food coupon worth $ 5 for each eligible person they recruited to the study). The RDS technique uses participants' social networks to access individuals who might not appear in public venues and might not be in contact with service providers. This technique has been proven to be effective for recruiting IDUs [27,28]. A face-to-face interviewer-administered structured questionnaire was used. In 2007 and 2009 the questionnaire was based on the WHO Drug Injecting Study Phase II survey (version 2b(rev.2)) [29], and in 2005, the questionnaire was adapted from several studies in resource-constrained and developed countries, including the Russian Federation [30,31]. Both instruments were originally developed to collect risk behavior data from IDUs, and therefore contained similar measures on key behavior characteristics (age, gender, age at injection initiation, injection frequency, main drug injected, receptive sharing within last 4 weeks, SEP utilization [ever, and in the last 4 weeks], number of sexual partners within last 12 months). The instruments were modified to obtain information on the illicit drugs known to be available in Tallinn.

Questions were selected that would elicit data on demographics, drug use history, HIV risk behavior, HIV testing, access and use of harm reduction services. A question on age at first injection was included, permitting calculation of the number of years the participant had been injecting. Interviews were held in confidence, in a room of the SEP. Recruitment was conducted and the survey administrated by a team of trained fieldworkers.

Blood was collected for HIV testing. In 2005 dried blood spot specimens were tested for HIV antibodies using GACELISA, reactive specimens were confirmed using an anti-HIV GACPAT immunoassay, with confirmatory testing conducted on discordant results using the HIV Blot 2.2 Western Blot assay (AbbotMurex) [32,33]. In 2007 and 2009, venous blood was tested with commercially available kits for HIV antibodies (Abbott IMx HIV-1/HIV-2 III Plus, Abbott Laboratories, Abbott Park, Illinois, USA).

Several studies comparing HIV antibody testing performance from serum (venous blood) and dried blood spots have documented excellent agreement of results between these different methods [34-36].

Data on the level of HIV prevention services provided were obtained from the Estonian National Institute for Health Development.

### Statistical analysis

HIV incidence can be estimated from cross-sectional data using prevalence data [37] stratified by time at risk (years of injecting) and assuming that HIV prevalence differences between the years of IDU duration strata represent incident HIV infections [10,20]. The questionnaires included questions on current age and age at first drug injection. Subtraction of age at first injection from current age provided a measure of the number of years injecting.

We estimated HIV incidence among new injectors using the following assumptions: (1) all of them were HIV seronegative when they began injecting; (2) the HIV seropositives became infected at the midpoint between beginning to inject and the time of blood sample collection, and (3) no HIV seropositives were lost to AIDS or for other reasons among the new injectors. We limited our analysis to "new injectors" (defined as a person reporting his/her first injection as occurring within three years of the study interview). For calculating time since first injection, we assigned persons who had first injected at their current age to have been injecting for 6 months, persons who first injected in the previous year to have been injecting for 1 year, persons who had first injected two years prior to their current age to have been injecting for 2 years, and persons who had first injected three years prior to their current age to have been injecting for 3 years. The time at risk for HIV seronegative new injectors is the total time from first injection to the time of the interview. The estimated HIV incidence rate was the number of HIV seropositive new injectors divided by the sum of the time at risk for the HIV seropositive new injectors (half the total time from beginning to inject to the time of interview) and the time at risk for the HIV seronegative new injectors (total time from beginning to inject to time of interview).

Study participation in all three studies was anonymous. To control for potential duplication in the samples (2005, 2007, 2009) a combination of biometric measures of each respondent (width of each wrist and length of each forearm from elbow to middle finger) and selected personal characteristics (sex, ethnicity, age) was used. Using this method, we identified one person who possibly participated in years 2005 and 2007. This person was retained for the analysis in both years, given the need for sufficiently large samples of new injectors, and that the HIV status, time at risk, use of syringe exchange, etc for that subject interviewed in 2005 would have reflected conditions in 2005, while if the same subject were interviewed in 2007, the subject's characteristics would primarily reflect conditions in 2006 in addition to reflecting conditions in 2007.

Risk behaviors and characteristics of 'new injectors' were compared in 2005, 2007, and 2009. Pearson's χ^2^, χ^2 ^test for trend and Fisher's exact tests were used for categorical variables. A multivariate analysis (controlling for age, sex, frequency of drug and SEP use) was used to test for trends in HIV prevalence [38].

RDS analysis Tool v. 5.0.1 was used to calculate homophily (the extent to which recruiters are likely to recruit individuals similar to themselves) to examine for possible recruitment bias [39].

Ethical approval was obtained from the Ethics Review Board of the University of Tartu, Estonia (in 2005 and 2009) and from the Tallinn Medical Research Committee (in 2007).

## Results

Table 1 shows the harm reduction services provided to IDUs in Tallinn from 2003 through 2009. The number of methadone treatment positions ("slots") increased from 49 to 209. With an estimated 10,000 IDUs in the city, however, the latest number of methadone treatment positions is still quite low, with methadone treatment available for only approximately 2% of the IDU population. Since 2004 the number of syringes distributed through SEPs (both stationary and outreach) in Tallinn has increased more than 6-fold. This corresponds to an estimated coverage of 70 or more syringes per IDU per year in 2008 (73.5 = 734,954/10,000) and 2009 (77.5 = 774,782/10,000).

We recruited 350 current IDUs in both 2005 and 2007 and 327 IDUs in 2009. In all three surveys, IDUs were predominantly male (80%), ethnic Russians (>80%), and young adults (mean ages 24 to 27 years) (full details from individual surveys are available from the first author). The proportion of new injectors (those injecting for less than three years) among the IDUs decreased from 21% in 2005, to 16% in 2007, and 12% in 2009 (p <0.005 by χ^2^test). Estimates for homophily indexes for the type of IDU (new and old injectors) in all three samples were close to zero, suggesting a single underlying population for each round of recruitment and non selective recruitment between the two IDU groups (i.e. new and old injectors) [27].

HIV prevalence among all subjects stabilized at slightly over 50% (54% in 2005, 55% in 2007, 50% in 2009). There were statistically significant differences in HIV prevalence among new injectors, with the prevalence in 2009 less than half that in 2007 (15.8% vs 34.2%) (Table 2). There were, however, differences in demographic characteristics and drug use behaviors among new injectors across the surveys. We used multiple regression [38] to determine if the differences in HIV prevalence remained statistically significant after controlling for age, gender, injection frequency and SEP use. The differences in HIV prevalence among new injectors remained statistically significant (Chi squared = 8.31, p = 0.016).

**Table 2:** New injectors, HIV prevalence and estimated incidence among new injectors (persons injecting less than 3 years) among surveys of IDUs in Tallinn, Estonia in 2005, 2007 and 2009

	The proportion of new injectors *			HIV prevalence **			HIV incidence per 100 PY ***	
**Year**	**New**	**All**	**%**	**HIV+**	**%**	**95% CI**		**95% CI**

**2005**	73	350	20,9%	25	34,2%	22.2-50.6%	20,9	13.5-30.8

**2007**	57	350	16,3%	22	38,6%	24.2-58.4%	26,5	16.6-40.1

**2009**	38	327	11,6%	6	15,8%	5.8-34.4%	9,0	3.3-19.6

* for changes of the period p = 0.005 (Chi-squared Test); ** for changes of the period p = 0.046 (Fisher Exact test); *** for changes of the period p = 0.026 (Chi-squared Test)

We also observed significant changes in the estimated HIV incidence among new injectors. In 2005 and 2007, the estimated incidences among new injectors were 20.9 per 100 person years (py), compared with 9.0/100 py in 2009 (Table 2).

During the period of the three surveys, there was an increase in the age of IDUs both in terms of chronological age and age of starting injecting (Table 3). Reported injection risk behaviors (any receptive sharing in the past 4 weeks) did not change over the three surveys. However, the proportion of new injectors reporting having used SEP services increased steadily throughout the study period (from 70% in 2005 to 97% in 2009), as did the proportion of new injectors for whom an SEP was the current main source of syringes (from 44% in 2005 to 76% in 2009). We did not see significant changes in reported numbers of sexual partners within the last 12 months or in condom use. There was a slight (but statistically not significant, p = 0.08) increase in the proportion of new IDUs who reported not always using condoms (37% in 2005, 58% in 2009).

**Table 3:** Socio-demographic and behavioural (risk) characteristics among new drug injectors in 2005, 2007, and 2009, in Tallinn, Estonia

	2005		2007		2009		P-value	
	**New injectors** **(N = 73)**		**New injectors** **(N = 57)**		**New injectors** **(N = 38)**		**Chi-squared Test**	**Chi-squared Test for Trend in Proportions**

	**N**	**%**	**N**	**%**	**N**	**%**		
**Socio-demographics**								
***Age (years)***								
< = 20	45	62%	23	40%	16	42%		
>20	28	38%	34	60%	22	58%	0.030	0.023
								
***Gender***								
Male	54	74%	45	79%	31	82%		
Female	19	26%	12	21%	7	18%	0.623	0.338
								
***Ethnicity***								
Russian+Russian speaking	57	78%	47	82%	34	89%		
Estonian	16	22%	10	18%	4	11%	0.330	0.140
								
**Environmental factors**								
***Ever in drug treatment***								
No	59	81%	48	84%	33	87%		
Yes	13	18%	9	16%	5	13%	0.799	0.503
								
***Ever in prison***								
No	44	60%	37	65%	26	68%		
Yes	29	40%	20	35%	12	32%	0.679	0.381
								
***SEP use ever***								
No	22	30%	16	28%	1	3%		
Yes	51	70%	41	72%	37	97%	0.003	0.003
								
***SEP as the main source of syringes (last 4 weeks)***								
No	41	56%	34	60%	9	24%		
Yes	32	44%	23	40%	29	76%	0.001	0.004
								
**Injecting drug use**								
***Age at IDU initiation (years)***								
< = 16	27	37%	7	12%	6	16%		
>16	46	63%	50	88%	32	84%	0.002	0.003
								
***Frequency of injection (last 4 weeks)***								
Less than daily	57	78%	30	53%	27	71%		
Daily	16	22%	27	47%	11	29%	0.008	0.189
								
***Main drug injected (last 4 weeks)***								
Fentanyl	35	48%	33	58%	23	61%		
Amphetamine	34	47%	23	40%	12	32%	0.321	0.132
								
***Receptive sharing (last 4 weeks)***								
No	54	74%	47	82%	30	79%		
Yes	19	26%	10	18%	8	21%	0.505	0.435
								
**Sexual behaviors**								
***Number of sexual partners (last 12 months)***								
0-1	32	44%	37	65%	15	39%		
1+	40	55%	20	35%	23	61%	0.022	0.984
								
Condom use (last 4 weeks)*								
always	26	36%	11	19%	9	24%		
else	27	37%	15	26%	22	58%	0.199	0.076

* Among those reporting vaginal or anal intercourses with in last 4 weeks

## Discussion

We have used a series of cross sectional studies conducted among IDUs over a period in which harm reduction services (mainly SEPs) were introduced and then expanded to examine the possible effects of such programs in an Eastern European country.

Several findings from our study are potentially important. The proportion of new injectors among IDUs across the surveys decreased; this suggests a decreasing rate of individuals becoming IDUs. Drug use patterns are not static, for example Estonia and several other Eastern European countries witnessed explosive IDU epidemics in the late 1990s. Limited data on the course of IDU epidemics after their emergence is available. In Russia, 47% of IDUs recruited in 2003 from Moscow, Volgograd and Barnaul [40] and 42% of IDUs recruited from St Petersburg in 2005-2006 [41] were new injectors (reporting injecting for <5 years). In Russia, 47% of IDUs recruited in 2003 from Moscow, Volgograd and Barnaul [40] and 42% of IDUs recruited from St Petersburg in 2005-2006 [41] reported injecting for <5 years. These findings are very similar to the 41% of IDUs recruited in Tallinn in our 2005 survey who reported injecting for <5 years. (In our 2005 survey 21% of subjects reported injecting for < = 3 years, see Table 2.) In Estonia, the decrease in numbers of new injectors occurred at the same time as the increase in SEP services. This contradicts the idea that providing SEP will lead to an increase in people beginning to inject drugs. Our sampling methods (RDS) did not change over the three surveys and no substantial changes of drug availability have occurred over the period under observation [25,42]. While the belief that SEP leads to more people beginning to inject drugs has generally been discredited [4,8], it is important to test this belief in middle income countries, where resistance to large-scale harm reduction remains strong.

We observed a significant decrease in the prevalence of HIV among new injectors from 34% and 39% in 2005 and 2007 to 16% in 2009. We also observed a decrease in the estimated HIV incidence among new injectors from up to 27/100 py in 2005 and 2007 to 9.0/100 py in 2009. The estimated incidence among new injectors showed a non-significant rise in 2007 (and a decline by 2009) coinciding with the rise and decline in the proportion of new injectors reporting daily injecting. While the level of HIV infection among new injectors in Estonia is still clearly unacceptably high, the change from the preceding years is substantial. Acknowledging that, in Estonia, active treatment for addiction (i.e. opioid substitution treatment) coverage and ART coverage among HIV infected IDUs [26] were implemented at a suboptimal level [43] during this period, preventing any meaningful public-health level effect, it is reasonable to attribute the decrease in HIV infection among new injectors to the greatly increased access to clean syringes/SEPs.

## Limitations

The cross-sectional design of the study sets well-known limits for causal inference. However, repeated cross-sectional studies may be considered to constitute a pseudo-longitudinal study, given that the IDUs recruited in the studies were sampled from the IDU source population using the same methodology. We used a non-probability sample that may have implications for the representativeness of the study results. However, RDS was used for recruitment because it is a sampling technique known to overcome some of the limitations of convenience sampling [27,28]. Other potential sources of bias associated with the sensitive and illegal behaviors under investigation are socially desirable responses and recall bias. However, we suggest that this would be expected to influence study participants in a similar way in different years. In addition, for HIV incidence estimations we made assumptions that might not hold. There is a possible loss of HIV seropositives from the active new injector population, due to ceasing to inject (perhaps because of methadone use), and to death/disability from AIDS. To minimize those effects we estimated the incidence only among new injectors, those who have injected for only a short period (3 years), and who would have been very unlikely to have developed AIDS and, given the limited availability of methadone treatment in Tallinn, they would also have been very unlikely to have received methadone treatment. It is of importance to note that we do not have data on the potential changes in IDU prevalence in Tallinn. However there are no declines in the numbers of criminal offences that can be related to the drug use or in the numbers of drug use related deaths in the recent years (2006-2009) [42].

It is also possible that the questions we used to measure injecting risk behavior did not capture important changes in injecting risk. For example, it is possible that IDUs in Tallinn reduced the frequency with which they shared injecting equipment (which would not be captured by the "any sharing" questions), or that they reduced the numbers of persons with whom they shared, or that some form of serosorting occurred, in which HIV seronegatives were more likely to share with other seronegatives and HIV seropositives were more likely to share with other seropositives. The numbers of syringes provided to IDUs may be a better measure of transmission probability than questions on "any sharing" during a short time period.

IDUs in Tallinn also obtain sterile injection equipment from pharmacies [28] and we were not able to determine the numbers of syringes obtained from pharmacies over the 2005 to 2009 time period. Despite some imprecision in determining the numbers of sterle needles and syringes obtained by IDUs in Tallinn over the time period covered by the surveys, it is clear from the data in Table 1 that there was a very substantial incorease in the provision of sterile injecting equipment. The coverage of 70 syringes per IDU per year reached by 2009 corresponds to the low coverage indicator set by the WHO, UNODC, UNAIDS for universal access to HIV prevention, treatment and care for injecting drug users [43], based on studies in the UK and Belarus [44] and the USA [45].

## Conclusions

Allocating resources for the prevention of HIV infection among IDUs is a challenging task. A large-scale SEP appears to have been quite effective in Estonia (a transitional country), although further reductions in HIV transmission among IDUs are still required. Coverage of 70 or more syringes per IDU per year may be needed before significant reductions in HIV incidence occur.

## Competing interests

The authors declare that they have no competing interests.

## Authors' contributions

AU, KAO, AT, KR and IS were responsible for and supervised the data collection in the individual studies. AU and DDJ planned the analysis for the manuscript; and MK conducted the statistical analysis. AU wrote the first draft of the manuscript. All authors contributed to revising the manuscript and have approved the final manuscript.

## Pre-publication history

The pre-publication history for this paper can be accessed here:

http://www.biomedcentral.com/1471-2458/11/517/prepub
